# A new hernia: Meckel’s diverticulum within a parastomal hernia

**DOI:** 10.1093/jscr/rjac398

**Published:** 2022-10-27

**Authors:** Mina Sarofim, Amir Ashrafizadeh, Shahrir Kabir

**Affiliations:** Department of Colorectal Surgery, Royal North Shore Hospital, Sydney, Australia; School of Medicine, University of New South Wales, Australia; School of Medicine, Sydney University, Australia; Department of Colorectal Surgery, Royal North Shore Hospital, Sydney, Australia; School of Medicine, Sydney University, Australia; Department of Colorectal Surgery, Royal North Shore Hospital, Sydney, Australia; School of Medicine, Sydney University, Australia

## Abstract

A Meckel’s diverticulum is a true diverticulum containing all layers of bowel wall. An infrequently encountered sequela of this is a Littre’s hernia, typically inguinal or femoral. This is the first description of a Meckel’s diverticulum within a parastomal hernia. When encountered incidentally at surgery, resection should be considered on a case-by-case basis to prevent future complications of bleeding, obstruction or perforation.

## INTRODUCTION

A Meckel’s diverticulum (MD) is an embryological remnant of the omphalomesenteric duct. An infrequently encountered sequela of this is a Littre’s hernia, typically inguinal or femoral. This is the first description of a Meckel’s within a parastomal hernia.

## CASE REPORT

**Figure 1:**
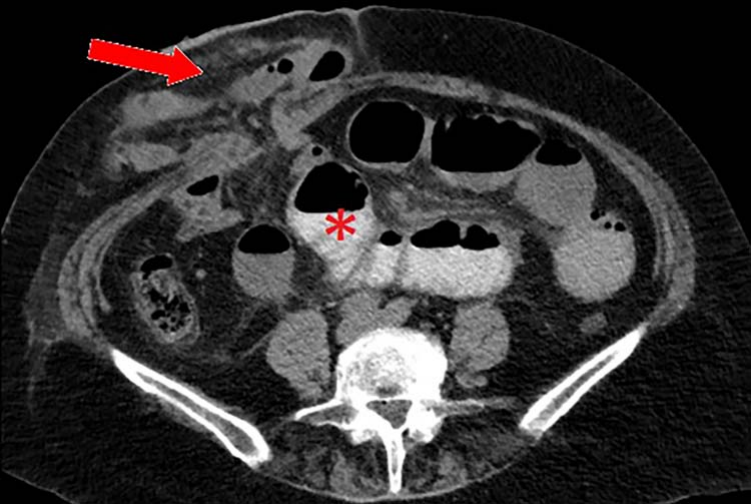
Axial CT scan with oral contrast demonstrating right-sided parastomal hernia containing small bowel loops (arrow) and proximal small bowel dilatation (asterisk).

We present a 71-year-old female admitted to a tertiary hospital for an elective repair of a parastomal hernia (loop ileostomy) for recurrent obstructive symptoms. Her background history was only significant for a severe long-standing colitis which necessitated defunctioning ileostomy 3 years prior. On examination, she had a partially reducible parastomal hernia containing loops of small bowel, confirmed on preoperative computed tomography (CT) imaging (Fig. 1).

Operatively, repair of the parastomal hernia was performed via a peristomal incision at the mucocutaneous junction of the loop ileostomy, followed by subcutaneous dissection and reduction of the hernia sac contents. A 4 cm MD was found on the ileal loops of bowel within the hernia sac, and simple diverticulectomy performed using a linear stapler. The anterior and posterior sheath were tightened around the stoma with 0 PDS, and a BD Phasix™ absorbable sublay mesh in keyhole fashion used to reinforce. The patient recovered uneventfully and discharged home on Day 5 post operatively.

## DISCUSSION

A MD is a true diverticulum containing all layers of bowel wall and an embryological remnant of the omphalomesenteric duct, as a result of incomplete obliteration during the fifth week of gestation. It is an uncommon finding ascribed to young doctors and medical students by the ‘rule of twos’: present in 2% of the population, typically symptomatic by age 2, 2 inches long, located 2 feet from the ileocaecal junction and often contains two types of ectopic mucosa (gastric and pancreatic) [1].

**Figure 2:**
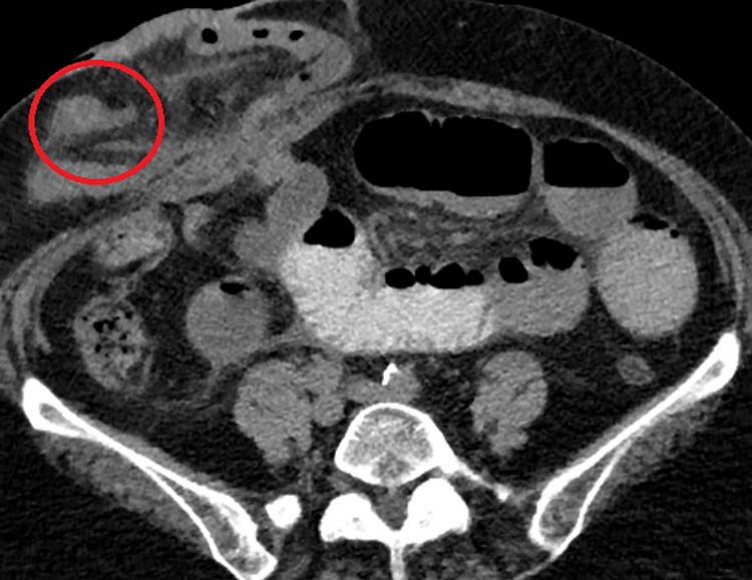
Axial CT scan demonstrating the MD (circled) arising from the anti-mesenteric aspect of the ileum within the parastomal hernia, identified after close retrospective review of imaging post operatively.

Clinical presentation of this diverticulum, if not asymptomatic, is gastrointestinal bleeding, bowel obstruction or inflammation/perforation [2]. Attempting to diagnose MD using CT imaging in patients with generic symptoms of abdominal pain or bleeding has an impressively low yield – often indistinguishable from adjacent loops of small bowel – thus a majority in adult patients are detected incidentally during surgery [3]. In the present case, after confirming the finding operatively, a close retrospective review of the CT identified the diverticulum (Fig. 2).

MD which herniates through the abdominal wall is called a Littre’s hernia. In a recent systematic review by Schizas *et al*. [4], the overall incidence of this hernia is exquisitely rare and affects only 1% of patients with MD. Sites of herniation are most commonly femoral (40%) or inguinal (34%), followed by infrequent umbilical, obturator or ventral hernias. There have been no documented MD within a parastomal hernia, and thus this represents the first description in the literature.

Symptomatic MD or those that incarcerate warrant resection, however there is some contention among surgeons with regard to management of those incidentally discovered intra-operatively. The decision to resect an incidental MD can only be made on an individual case-by-case basis – the potential morbidity of a diverticulectomy or formal small bowel resection must be balanced against the risk of future complications if left in situ. Age under 50, male gender, diverticulum longer than 2 cm, palpable abnormality or association with an abdominal band predispose to symptoms, and surgeons should opt to resect in these situations [2, 5]. Although detected incidentally in the present case, the length and palpable abnormal nodularity warranted resection.

With regard to parastomal hernia, they are known to occur within the first 2 years after stoma formation with an incidence of up to 40% [6]. These hernias are most commonly managed conservatively but surgical repair (revision, resiting or reversal) is indicated if there is unmanageable leakage due to ill-fitting appliances, severe pain, cosmetic concerns or acute strangulation [7]. Primary suture repair is considered outdated due to recurrence rates close to 70%. Mesh repair offers more acceptable recurrence rates ranging from 7 to 18%; sublay or intraperitoneal placement (such as the Sugarbaker repair) reported as superior to onlay with no significant increase in peri-operative complications [8].

A Littre’s hernia is an infrequently encountered sequela of a MD, typically inguinal or femoral. This is the first description of a Meckel’s within a parastomal hernia and when encountered incidentally at surgery, resection should be considered on a case-by-case basis to prevent future complications of bleeding, obstruction or perforation.
